# Can Fasting Curb the Metabolic Syndrome Epidemic?

**DOI:** 10.3390/nu14030456

**Published:** 2022-01-20

**Authors:** Josip Vrdoljak, Marko Kumric, Marino Vilovic, Dinko Martinovic, Veljko Rogosic, Josip A. Borovac, Tina Ticinovic Kurir, Josko Bozic

**Affiliations:** 1 Department of Pathophysiology, University of Split School of Medicine, 21000 Split, Croatia; josip.vrdoljak@mefst.hr (J.V.); marko.kumric@mefst.hr (M.K.); marino.vilovic@mefst.hr (M.V.); dinko.martinovic@mefst.hr (D.M.); jborovac@mefst.hr (J.A.B.); tticinov@mefst.hr (T.T.K.); 2 Department of Ophthalmology, University of Split School of Medicine, 21000 Split, Croatia; veljko.rogosic@mefst.hr; 3 Department of Ophthalmology, University Hospital of Split, 21000 Split, Croatia; 4 Department of Health Studies, University of Split, 21000 Split, Croatia; 5 Department of Cardiology, University Hospital of Split, 21000 Split, Croatia; 6 Department of Endocrinology, Diabetes and Metabolic Diseases, University Hospital of Split, 21000 Split, Croatia

**Keywords:** metabolic syndrome, fasting, intermittent fasting, time restricted feeding, diabetes mellitus, hypertension, dyslipidemia, obesity

## Abstract

Metabolic syndrome (MetS) represents a cluster of metabolic abnormalities that includes hypertension, central obesity, insulin resistance, and atherogenic dyslipidemia. Due to the high prevalence (around 1/3 of the world population) economic burden of MetS, there is a need for new dietary, lifestyle, and therapeutic options. Recently, fasting emerged as a dietary method proposed for controlling metabolic risk factors. Intermittent fasting (IF), or time-restricted feeding (TRF), describes an array of feeding patterns in which calorie intake is restricted to a specific time period. Hence, this review aimed to elucidate the latest data on MetS and explore the viability of simple management options, such as IF and TRF. Preclinical studies have shown how IF/TRF exerts beneficial effects on the gut microbiota, glucose and insulin metabolism, weight and visceral fat, and lipid metabolism. However, the results obtained from human studies are somewhat conflicting, as weight loss was achieved in all studies, whereas in some studies, there was no significant effect on insulin resistance, cholesterol/lipid metabolism, or blood pressure. Nevertheless, as only very few human studies were performed, there is a need for more randomized control trials on larger cohorts of patients with MetS to gather higher-yield evidence to clarify whether IF/TRF are suitable dietary patterns for this population.

## 1. Introduction

Central obesity, elevated blood pressure, dyslipidemia, and insulin resistance are cardiometabolic risk factors grouped together in metabolic syndrome (MetS). Since the WHO defined MetS in 1999, there have been multiple other definitions of MetS, with the most recent harmonized definition [1,2]. All components of MetS are plaguing the world with an increasing incidence and prevalence [3,4,5]. Obesity, mostly of the central type, is a pathophysiological cornerstone of MetS and is considered to be the main culprit that leads to other metabolic disturbances found in MetS [4,6,7,8]. Epidemiological studies have shown that in the last 40 years, obesity prevalence has doubled, to such a degree that nearly a third of the world’s population is now considered overweight or obese [5,9]. Before the start of the globalization trend, obesity was most prevalent in the US and the countries of Western Europe, while currently we are witnessing an increase in obesity rates in all ages and sexes, regardless of geographical location, socioeconomic status, and ethnicity [5,10]. Likewise, the prevalence of MetS in the US is also high, at 34.7% (95% CI, 33.1–36.3%) [11]. Similar trends in prevalence are also found in other countries across the globe, with the highest MetS prevalence in Mexico (41%) [12,13,14,15].

Furthermore, the prevalence of hypertension is also on the rise, particularly in low-middle income countries [16,17,18]. In addition, the same is true for the prevalence of insulin resistance/diabetes mellitus type 2 and dyslipidemia [19,20]. The cost of treating these comorbidities is substantial. According to the American Diabetes Association (ADA), the total costs of diagnosed diabetes have risen from $245 billion in 2012 to $327 billion in 2017, resulting in a 26% increase over a five-year period [21]. Moreover, adults with obesity in the US experienced $2505 higher annual health care costs, which is a 100% higher cost paid compared to those with lesser weight [22]. Similarly, according to American Heart Association (AHA) and Medical Expenditure Panel Survey (MEPS) data from (2016–2017), the costs of treating cardiovascular disease (CVD) in the US were estimated to be $363.4 billion (or $406 billion, inflation-adjusted) [23].

There are no studies directly examining the economic burden of MetS per se. Still, since other studies have shown how the prevalence of MetS among obese adolescents and adults ranges from 30.3% up to 52%, we may postulate that the direct medical costs of MetS are around 1/3 to 1/2 of the costs of obesity (from $78 billion to $135 billion) in the US [24,25]. Since MetS and obesity mostly precede CVD and type II diabetes, there is a growing need for cheap and effective treatment strategies to combat this increasing burden.

Lifestyle and dietary changes (physical activity, calorie intake/bodyweight reduction) are at the forefront of metabolic syndrome treatment [26,27,28]. One of the dietary methods proposed for controlling metabolic risk factors is fasting [29]. Intermittent fasting (IF) and time-restricted feeding (TRF) describe an array of feeding patterns in which calorie intake is restricted to a specific time period (8 h feed/16 h fast, 4 h feed/20 h fast, alternate day fasting, etc.) [30]. Recently, IF/TRF has shown promising results in managing cardiometabolic risk factors [29,31,32].

Hence, in the first part of this comprehensive review, we present the latest data on MetS epidemiology and pathophysiology, while in the second part, we explore the viability of IF and TRF for managing MetS.

## 2. The Definition of Metabolic Syndrome

In 1988, during his Banting lecture, Reaven characterized a cluster of conditions related to insulin resistance. He called the cluster “Syndrome X” or “the deadly quartet”, and it consisted of obesity, non-insulin-dependent diabetes mellitus, hypertension, and dyslipidemia [26,33]. Since then, multiple definitions have been brought forward to better encompass this clustering of cardiometabolic risk factors. These include the WHO definition from 1998, the NCEP (National Cholesterol Education Program) definition from 2003, and the IDF (International Diabetes Federation) definition from 2006 [20]. To avoid further discrepancies and to standardize the diagnostic criteria, several major health organizations jointly produced the harmonized definition of MetS that is currently in use [34]. 

According to the harmonized definition, MetS is defined by the following criteria:

(1) The presence of insulin resistance/prediabetes (glucose level > 100 mg/dL (5.6 mmol/L)), or diagnosed type 2 DM. 

(2) Enlarged waist circumference (the exact values of which are adjusted according to population-specific and country-specific criteria).

(3) HDL-C < 40 mg/dL (1.03 mmol/L) in men and <50 mg/dL (1.29 mmol/L) in women or triglycerides ≥ 150 mg/dL (1.69 mmol/L) (with the inclusion of those taking medicine to treat dyslipidemia). 

(4) Systolic blood pressure ≥ 130 mm Hg or diastolic blood pressure ≥ 85 mm Hg (including patients on anti-hypertensive therapy).

## 3. Epidemiology of Metabolic Syndrome

The central type of obesity is the most common characteristic found in MetS; therefore, the incidence and prevalence of MetS closely follow that of obesity [35,36]. Every two years, the US conducts the National Health and Nutrition Examination Survey (NHANES), in which they obtain obesity rates among people aged two or older. The latest data for 2017–2018 show that obesity prevalence among adults was 42.4% [37]. By contrast, the obesity rates for adults at the start of the decade were 35.7%, with a rising trend of approximately 2% every two years. The rising trend is slower for the pediatric population, with the 2009–2010 obesity rate at 16.9% and the 2017–2018 obesity rate at 19.3% [37]. In a study by Hirode et al., where the authors examined NHANES data form 2011 to 2016, among 17048 participants, the MetS weighted prevalence was 34.7% (95% CI, 33.1–36.3% [*n* = 5885]) [11]. In other words, as much as one-third of the US adult population suffers from MetS. 

The large prevalence is not restricted to the US, as comparable data were found in Brazil, where the latest MetS prevalence was 38.4% [13]. High waist circumference (65.5%) and low HDL cholesterol (49.4%) were the most prevalent MetS components among the Brazilian population. In addition, MetS was more frequent among women (41.8%), individuals with less education (47.5%), and older adults (66.1%) [13]. Similarly, in Mexico, investigators performed a systematic meta-analysis on 15 studies in which the pooled prevalence of MetS was 41% (95% CI 0.34–0.47) [12].

In Asia, a metanalysis in which investigators pooled MetS prevalence data from the Chinese population from 2008–2015 found that the pooled prevalence for subjects aged 15 years and older was 24.5% (95% CI: 22.0–26.9%). This metanalysis on the Chinese population also saw a similar trend, according to which the MetS prevalence was higher in females 27.0% (95% CI: 23.5–30.5%) vs. males 19.2% (95% CI: 16.9–21.6%) [38]. However, another study on the Chinese population showed marked differences in MetS prevalence between various ethnic groups. The Korean population featured the highest MetS prevalence (35.42%), the Hui population the second highest (22.82%), while the Mongolian and Tibetan populations featured the lowest (11.61%) and (6.17%) respectively [39]. 

Furthermore, in a European study examining data from two cohorts, one from Russia and the other from Italy, the MetS prevalence was 37% for the former and 21% for the latter [40]. In addition, another study examined the data from 34,821 subjects from 12 cohorts from 10 European countries and one cohort from the USA. MetS prevalence was 24.3% (8468 subjects: 23.9% in men vs. 24.6% in women, *p* < 0.001), with an age-related increase in prevalence across all cohorts [15]. Furthermore, in a study on the Portuguese population, in which data were gathered from 2007 to 2009, MetS prevalence was 36.5%, 49.6%, and 43.1%, using the Adult Treatment Panel III, International Diabetes Federation, and Joint Interim Statement definitions, respectively [14]. MetS prevalence was significantly higher in women and the older population in Portugal, as shown in the aforementioned studies. At the same time, it was also more frequent in non-urban areas than in urban areas (*p* = 0.001) [14]. Interestingly, in contrast to these findings, a study on the Czech population found that MetS is less common in females 25.5%, then in males 37.6% [41]. Akin to these findings, MetS prevalence was also higher in Slovakian males (30.2%), than in females (26.6%), with an increasing trend from 2003 to 2012 [42]. 

When examining the data we mentioned, we can see large differences in MetS prevalence among various populations, from the low of 11.61% in the Tibetan population, to the high of 41% in the Mexican population [12,39]. The reasons behind the observed discrepancies are probably a result of different lifestyles, with Western dietary habits making people more susceptible to MetS. If we take a closer look at migrant populations (from countries with low to countries with high rates of obesity) we observe a rather interesting twist. Namely, although migrants arrive with a health advantage, including generally healthier body weight, intrinsic and environmental factors combine to cause unhealthy weight gain, often to beyond the levels seen in native populations [43]. According to Neel et al., this observation may be explained from an evolutionary standpoint. Although the loss of uricase may have provided a survival advantage by amplifying the effects of fructose to enhance fat stores, and by increasing blood pressure in response to salt, the absence of the “thrifty” uricase gene may have caused a range of detrimental metabolic effects on modern humans (characterized by excessive caloric intake), thus explaining the current epidemic of obesity and diabetes [44]. The putative mechanism associating fructose and loss of uricase lies in the observation that uric acid may regulate fructose metabolism by affecting fructose transporters in the intestines and fructokinase in the liver [45]. Nevertheless, with the Western lifestyle prevailing in most of the world, there is an increasing global trend of MetS prevalence, with approximately one-quarter of the world’s population currently suffering from MetS [11,20,46].

## 4. Pathophysiological Background of Metabolic Syndrome

As is the case with other chronic non-communicable diseases, MetS also results from a complex interplay between genetic and environmental factors. Currently, central obesity/visceral adipose tissue (VAT) is considered to play one of the main roles in initiating the deadly quartet of MetS. VAT exerts its influence on glucose and lipid metabolism via multiple mechanisms. Firstly, VAT is a major source of free fatty acids (FFA), which are directly connected to the liver via splanchnic circulation [4]. In the liver, FFAs lead to increased gluconeogenesis, as well as increased triglycerides and very low-density lipoprotein (VLDL) production [6]. The increase in liver FFA oxidation induces a decrease in xylulose 5-phosphate, which results in the activation of gluconeogenesis (by inhibiting phosphofructokinase 1 and activating fructose-1,6-bisphosphatase) [47]. Furthermore, the ectopic accumulation of lipid metabolites (ceramides, diacylglycerol, acetyl-CoA and fatty acids) decreases insulin sensitivity [48]. These lipid metabolites, in turn, activate serine/threonine kinases (protein kinase C (PKC), nuclear factor-kB (NFkB), inhibitory kB kinase b (IKKb)), which then phosphorylate insulin receptor substrate (IRS) and protein kinase B/Akt, and therefore inhibit insulin signaling [47,48]. 

Moreover, adipose tissue is a source of many cytokines and hormones, called adipokines. Studies have shown that central obesity/VAT is related to dysregulated adipokine secretion, with increased levels of plasminogen activator inhibitor (PAI-1), tumor necrosis factor-alpha (TNF-α), monocyte chemotactic protein-1 (MCP-1), angiotensinogen, and interleukin 6 (IL-6). In addition, leptin, a hormone that regulates satiety, energy expenditure, and appropriate glucose homeostasis, is directly correlated to the amount of white adipose tissue. Even though, in physiological conditions, leptin promotes satiety and signals the amount of fat storage to the hypothalamus, it seems that in MetS, there is a leptin resistance or a certain ceiling on the possible effect of leptin, beyond which new leptin stimulates little effect [49]. 

On the other hand, in central obesity, there are decreased levels of adiponectin, which is considered the “good” adipokine. The primary action of adiponectin is phosphorylation and the activation of key intermediates in the insulin signaling pathway, increasing insulin sensitivity [50]. Therefore, a lack of adiponectin in MetS promotes insulin resistance and disrupts glucose homeostasis. 

The aforementioned TNF-α, IL-6, and MCP-1 are pro-inflammatory cytokines, which contribute to the systemic low-grade inflammation found in MetS. At the same time, PAI-1 increases the risk of thrombosis and accelerates the development of atherosclerosis [4,51]. This low-grade inflammation leads to further insulin resistance in muscles, as well as to disruption of o β-cells [52,53]. 

As recently reviewed, there is also essential gut–adipose tissue crosstalk, which is disrupted in the setting of MetS. Postprandial incretins, glucagon-like peptide 1 (GLP-1), and glucose-dependent insulinotropic peptide (GIP), which regulate glucose homeostasis and exert anorexigenic effects, are significantly decreased in obese or T2DM patients [54]. Furthermore, in patients with obesity and MetS, the levels of appetite-inducing hormone ghrelin fail to progressively decline after meal ingestion [54]. This disruption in anorexigenic/orexigenic hormone homeostasis induces a positive loop that ultimately ends in obesity/MetS.

Finally, the pathogenesis of hypertension in MetS is multifactorial as well. Hyperinsulinemia exerts an anabolic effect on the heart muscle and the media of the blood vessel wall. It also promotes sympathetic nervous system (SNS) and renin-angiotensin-aldosterone system (RAAS) activity, leading to vasoconstriction, sodium retention, and endothelial dysfunction [55]. Interestingly, recent studies have also elucidated leptin’s role in obesity-related hypertension. Acting on its receptors in the hypothalamus, leptin initiates a downstream signal transduction that ends in the preganglionic autonomic neurons of the spinal cord, leading to increased sympathetic activity in the kidneys and, therefore, increased blood pressure [56]. Nevertheless, each presented mechanism’s relative contribution to hypertension development remains elusive.

Moreover, the presence of obstructive sleep apnea and baroreflex dysfunction in MetS further increase SNS activity [57,58,59]. 

While the current understanding of MetS pathophysiology is discussed above, it is a continuously improving subject with new research that will help us better understand the puzzle of MetS.

## 5. Effects of Diet on Metabolic Syndrome

The Western diet, characterized by a high intake of red and processed meat, refined grains, sweets, and sugary beverages, is associated with an increased risk of developing MetS [60,61]. This diet is calorie-dense, rich in small-chain fatty acids (SFA), simple carbohydrates, and other nutrients that feature pro-inflammatory properties, disrupt the gut microbiota, and dampen insulin sensitivity [61,62,63]. A meta-analysis by Fabiani et al. has shown that the “Meat/Western” pattern leads to a 19% increase in MetS risk, while a “Healthy” dietary pattern (fruit, vegetables, whole grains, fish, no processed food/high content of vitamins, minerals, antioxidants, fiber, MUFA, and n-3 fatty acids) is associated with a 15% decrease in MetS risk [60]. Similar results were obtained in another meta-analysis that also studied the relationship between a posteriori dietary patterns and MetS: a healthy/prudent diet was associated with a lower prevalence of MetS, while an unhealthy/Western pattern was associated with an increased risk of developing MetS [64]. Another popular dietary pattern, the Mediterranean diet, has also shown benefits regarding MetS [65,66]. In a meta-analysis by Kastorini et al., the combined effect of prospective studies and clinical trials showed that the Mediterranean diet is associated with a reduced risk of MetS (log hazard ratio: −0.69, 95% confidence interval (CI): −1.24 to −1.16) [67]. Comparable results were achieved with the dietary approaches to stop hypertension (DASH), where multiple studies showed how the DASH diet led to a reduction in systolic and diastolic blood pressure, a reduction in BMI and waist circumference, an improvement in cardiometabolic profile, and a reduction in T2DM incidence [68,69,70,71,72].

Therefore, there is substantial evidence that diets such as the Mediterranean diet and DASH exert a beneficial effect on cardiometabolic risk factors, with a common theme in which foods such as vegetables, fruit, whole grains, and fish are associated with these benefits. Nevertheless, in addition to the diet itself, dietary regime adjustments may provide metabolic benefits regardless of the amount and type of food ingested.

## 6. Metabolic Syndrome and Fasting

### 6.1. Evidence on Animal Models

A number of studies have exhibited the beneficial effects of fasting/time-restricted feeding on animal models. A number of studies have exhibited the beneficial effects of IF/TRF on animal models. In a study by Hatori et al., the authors demonstrated the favorable effects of TRF in mice fed with a high-fat diet (HFD, 61% energy from fat). The group fed during an 8 h period lost weight and exhibited better insulin sensitivity, less hepatosteatosis, less inflammation, and improved motor coordination while consuming the same number of calories as the group fed ad libitum (AL) [73]. The beneficial effect of TRF was substantiated from a biochemical standpoint as well, since the TRF regimen improved CREB, mTOR, and AMPK (metabolic master regulator) pathway function [73]. Similar results were achieved in another study where, in comparison to mice on high-fat (HF) AL diets, mice on a time-restricted HF diet experienced an 18% reduction in body weight, a 30% reduction in cholesterol levels, 10% lower TNF-α levels, and a 3.7 fold increase in insulin sensitivity [74]. Moreover, in the same study, the authors compared mice on a time-restricted HF diet with mice on an AL low-fat diet consuming the same number of calories. In this comparison, the TRF mice showed a 2% bodyweight reduction, a 21% reduction in cholesterol levels, and a 1.4 fold increase in insulin sensitivity [74].

Furthermore, in a recent study, Pak et al. used a series of feeding regimens to dissect the effects of caloric restriction (CR) and fasting [75]. Four groups of mice were examined: (1) mice given AL access to a regular rodent diet; (2) mice provided with AL access to chow that was 50% diluted with indigestible cellulose (corresponding to a 30% CR); (3) mice fed 30% less food than AL-fed mice using an automatic feeder to release food in three equal portions during the 12 h dark period; (4) mice fed once per day in the morning, with 30% restriction compared to the AL group; (5) mice that were trained to consume approximately the same quantity of the food as AL-fed mice, but within three hours. The crucial comparison was between mice on a diet according to which they consumed fewer calories without imposed fasting (AL chow diluted with 50% indigestible cellulose) and mice that were fed only once per day in the morning (without CR). The study showed that fasting is needed for CR-induced improvements in glucose metabolism, frailty, and lifespan in C57BL/6J male mice. In addition, the results elucidate how fasting alone, without the reduced intake of calories, is sufficient to realize the metabolic phenotypes and transcriptional signature of a CR diet [75]. The importance of these studies that they show the benefits of TRF whilst controlling for caloric intake, thus decoupling the effects from caloric restriction.

Other studies have proven the benefits of fasting in alleviating circadian clock disruptions and improving metabolic homeostasis [29,76]. As reviewed in a paper by Świątkiewicz et al., a proper TRF cycle sustains circadian rhythms and restores normal daily rhythms in many mRNAs, proteins, and metabolites that are involved in the homeostasis of carbohydrates and lipids [29]. TRF also regulates circulating leptin and adiponectin levels, and exerts multiple valuable effects on the main organs involved in metabolic homeostasis (liver, muscle, white and brown adipose tissue, gut, and brain) [29,76,77,78]. Interestingly, a study on mice has shown the effects of IF (every-other-day fasting – EOFD) on white adipose tissue browning [79]. The EOFD mice exhibit significantly more beige fat development within white adipose tissue, were less obese, better insulin sensitivity, and less hepatic steatosis. The researchers also pointed out how these beneficial effects are most probably exerted through the shaping of gut microbiota because the microbiota-depleted mice were resistant to EODF-induced beiging, while microbiota transplantation from EODF-treated mice to microbiota-depleted mice activated beiging and improved metabolic homeostasis [79]. Moreover, a study on a drosophila melanogaster (fruit fly) model of obesity, in which flies were subjected to a 12 h TRF, exhibited improved muscular function (fewer intramuscular fat deposits), improved phosphorylated AKT levels, fewer mitochondrial aberrations, and better insulin sensitivity [80].

Importantly, not all fasting protocols exhibit beneficial effects, as was shown in a study investigating alternate-day fasting in young female Wistar rats (24 h fasts intercalated with 24 h of free access to the same chow) [81]. Alternate-day IF decreased weight gain and food intake, but it led to increased fat reserves, elevated plasma insulin concentrations, and reduced muscle mass. Although the study was conducted on young and healthy animals with no MetS, these findings suggest the potential adverse effects of alternate-day fasting and promote caution in thinking that all fasting methods are similar and beneficial.

**Figure 1:**
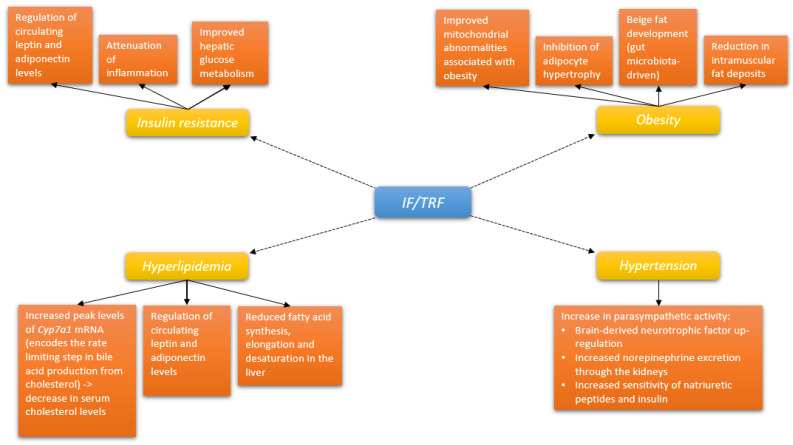
Pathways through which intermittent fasting/time restricted feeding may affect the constituents of metabolic syndrome. Abbreviations: IF: intermittent fasting; TRF: time-restricted feeding.

Overall, the above-noted animal studies have set the biochemical and pathophysiological evidence and groundwork (positive effects on weight, obesity, gut microbiota, energy metabolism, and circadian rhythms) for further human studies on patients with obesity and MetS (Figure 1, Table 1).

### 6.2. Evidence in Human Studies

Currently, there is little research on the effects of fasting on patients with diagnosed MetS [29,31]. However, several small-scale and pilot trials on obese patients and patients with cardiometabolic risk clustering (but no MetS diagnosis) have provided promising results [29,31,82]. Similarly, a recent systematic review on the effects of fasting on cardiometabolic risk factors supports the role of IF in improving metabolic health [83]. Small but significant improvements were detected in risk factors such as body weight, waist circumference, fat mass, BMI, blood pressure, total cholesterol, triglycerides, fasting blood glucose, fasting insulin, and insulin resistance [83].

On the other hand, there are also some contradictory results, as one randomized control trial on obese or overweight adults produced only modest weight loss (below 2% of initial body weight), but no real benefits of IF on metabolic parameters or fat loss in the absence of controlled food intake [84].

A single-arm, paired-sample trial by Wilkinson et al. investigated the effects of 14 h fasts for 12 weeks on 19 patients with MetS who received the standard of care (antihypertensive therapy and/or statin) [31]. To track caloric intake and the adherence to TRE intervention, a validated app—myCircadianClock (mCC) was used [31]. Although there were no recommendations to reduce caloric intake, an 8.62% decrease in mean daily caloric intake was recorded during the intervention. Significant bodyweight reduction from baseline was achieved (−3%), along with desirable reductions in body fat percentage (−3%), along with significant decreases in visceral fat rating (−3%) and waist circumference (−4%) [31]. Importantly, the authors also noted significant decreases in systolic and diastolic blood pressure, total cholesterol, LDL-C, and non-HDL-C [31]. Despite the study’s limitations (it was an unblinded, single-arm pilot study with a relatively small sample size), it still provided valuable evidence on the safety, adherence, and probable efficacy of TRF in MetS management.

In a proof-of-concept study, Sutton et al. investigated the effects of 5 week early TRF (eTRF, eating period from 8 a.m. to 2 p.m.) in men with prediabetes, while controlling for weight loss (feeding the participants enough food to maintain their weight) [84]. In the study, eTRF reduced insulin levels, blood pressure, and oxidative stress, while it improved β-cell responsiveness and insulin sensitivity. Although the study was conducted on just eight participants, excellent adherence was achieved, since the participants were required to eat all meals under supervision [84,85]. The importance of this randomized controlled trial was that it demonstrated the benefits of IF independent of food intake and weight loss in humans.

In another study, researchers examined the effects of 8 h TRF over 12 weeks on 23 obese adults, compared to a matched historical control group [82]. There were significant decreases in body weight, energy intake, and systolic blood pressure in the TRF group (−2.6% ± 0.5; −341 ± 53 kcal/d; −7 ± 2 mm Hg, respectively). Interestingly, there were no significant differences in fat mass, lean mass, visceral fat mass, diastolic blood pressure, LDL cholesterol, HDL cholesterol, triglycerides, fasting glucose, fasting insulin, HOMA-IR, or homocysteine [82].

Kesztyüs et al. conducted a pilot study on the effects of TRF in abdominally obese participants (waist-to-height ratio, WHtR ≥ 0.5) in a general practitioner’s office [86]. Forty participants were asked to restrict their daily eating time to an 8–9 h window. On average, the participants reached the 15–16 h fasting target in 86 ± 15% of all recorded days. In addition, the participants achieved moderate weight loss (−1.7 ± 2.5 kg), along with a marked reduction in waist circumference (−5.3 ± 3.1 cm), leading to a reduction in WHtR (−0.03 ± 0.02) [86].

Moreover, a randomized controlled trial that researched the effects of two-month 4 and 6 h TRF in obese adults, resulted in promising outcomes concerning weight and cardiometabolic health [87]. Of 58 participants, 19 were randomized into the 4 h TRF group, 20 into the 6 h TRF group, and 19 into the no-intervention control group. Both the 4 and 6 h TRF regimens achieved a similar reduction in daily calorie intake (−550 kcal), while also producing similar weight loss (−3% body weight). Additionally, they reported a marked reduction in fasting insulin, insulin resistance, and oxidative stress. However, the significance of insulin and insulin resistance reductions was partly driven by a worsening in the control arm [87].

A trial by Parr et al. investigated the feasibility of TRF for individuals with T2DM [88]. The intervention consisted of a 2 week Habitual period to establish a baseline dietary intake, followed by a 6 week TRF intervention. Of the 24 enrolled participants, 19 completed the study. Overall daily dietary intake did not change between habitual and TRF periods. Moreover, the compliance with the 9 h TRF period was 72 ± 24% of 28 days (i.e., ~5 days/week). Interestingly, TRF did not significantly influence glycemic control or body mass, whereas the participants described hunger, daily stressors, and emotions as the main barriers to adherence. [88]. Furthermore, in a study by Chow et al., the authors examined and compared the effects of TRF (8 h target eating window) to an unrestricted (non-TRF) diet in overweight individuals [89]. Compared to non-TRF, the TRF group exhibited significantly reduced weight, lean mass, and visceral fat. Furthermore, when the TRF group was compared to their pre-intervention state, marked reductions in weight (−3.7%), fat mass (−4%), lean mass (−3.0%), and visceral fat (−11.1%) were observed. Interestingly, metabolic measures (lipids, blood pressure, 2 h oral glucose tolerance test, 2 week continuous glucose monitoring), and physical activity (actigraphy-assessed) remained unchanged [89]. The same research group performed a secondary analysis of the aforementioned trial, where they examined the effects of TRE on quality of life (QoL) measures [90]. TRE did not adversely affect QoL, and it even led to modest QoL improvements relative to baseline and unrestricted eating [90].

By contrast, in a prospective, randomized controlled trial conducted on 116 overweight or obese adults, Lowe et al. compared the effects of TRF (eating from 12–8 pm) with consistent meal timing (CMT-3 structured meals per day) over 12 weeks [84]. There was no recommendation for calorie and macronutrient intake or physical activity, so the study only compared the effects of different meal timing. There was a significant decrease in weight in the TRF group (−0.94 kg; 95% CI, −1.68 kg to −0.20 kg; *p* = 0 .01), but no significant differences in weight change between groups (−0.26 kg; 95% CI, −1.30 kg to 0.78 kg; *p* = 0.63). There were no significant within-group or between-group differences in glucose and lipid metabolic parameters [84].

In a randomized controlled trial, Parvaresh et al. investigated the differences between a modified alternate-day fasting regime (ADF) (a very low-calorie diet, 75% energy restriction on Saturday, Monday, and Wednesday accompanied by 100% energy intake on Sunday, Tuesday, Thursday, and ad libitum feeding on Friday) and a standard caloric restriction diet (consuming 75% of energy needs each day) during an 8 week period [91]. Of 70 patients with MetS, 69 completed the study, and the analysis showed significant reductions in body weight, waist circumference, systolic blood pressure, fasting plasma glucose in the ADF group. Interestingly, there were no significant differences in triglyceride, cholesterol (total, HDL, LDL), HOMA- IR, or fasting insulin concentrations [91]. The strengths of this study are in the number of participants (69, compared to the 23 in the second-largest study on MetS/fasting) and its randomized design [31,91].

In summary, a common theme in all of the aforementioned studies is a significant reduction in body weight and waist circumference (Table 2). At the same time, there are mixed results regarding the improvement in insulin sensitivity, glucose and lipid homeostasis, and blood pressure. Interestingly, there are only two studies conducted on participants with diagnosed MetS. In addition to significant weight and waist circumference reduction, both studies also reported a significant reduction in blood pressure levels, albeit with mixed results in cholesterol reduction [31,91]. However, it is possible that the observed improvements using IF/TRF may not be sustained unless close monitoring and follow-up of adherence are performed. Therefore, a combination of patient education with dietary, medical, and coaching staff familiar with the IF protocol is critical in achieving the putative results. On the other hand, the potential downsides of IF/TRF that require attention are hypoglycemic reactions, cardiac arrhythmias, muscle wasting, menstrual irregularities, gout, postural hypotension, peptic ulcers, and upper gastrointestinal bleeding [92,93]. As expected, hypoglycemic reactions represent the main issues among patients with diabetes, especially in T1DM [92].

## 7. Conclusions and Future Perspectives

Due to the high MetS prevalence (around 1/3 of the world population), its increasing trend, and high economic burden ($260.6 billion aggregate costs due to obesity in the US), we are in dire need of new dietary, lifestyle, and therapeutic options [3,94]. Diets such as the Mediterranean diet and DASH have proven beneficial, but quality healthy foods such as fruit, vegetables, and fish are more expensive, and hence less accessible to a large part of the population [95,96]. Therefore, diets such as fasting/TRF that do not necessarily need a change in diet quality (with expensive foods) to exert beneficial results could prove valuable in combating this epidemic. A significant amount of evidence was gathered on the efficacy of IF/TRF in animal models [73,74,75,76,78]. These studies showed how IF/TRF exerts beneficial effects on the gut microbiota, glucose and insulin metabolism, weight and visceral fat, and lipid metabolism. In other words, fasting affects all the crucial pathophysiological points in MetS/diabetes type II development [4]. However, it is important to acknowledge that different species and the different ages of the tested animals affected the response to IF/TRF, and this variability should therefore be taken into account when assessing metabolic effects of IF/TRF. Considering studies on humans, while the results are promising, they are still scarce (there are only two studies on patients with diagnosed MetS). Most of the studies were performed on overweight/obese individuals who often present at least one more cardiometabolic risk factor, so we can expect similar results in larger studies on MetS patients. It is important to note that the viability and safety of IF/TRF methods have been established in small-scale, pilot studies [31,82,85,89]. The results obtained from human studies are somewhat conflicting, as weight loss was achieved in all studies, whereas in some studies, there was no significant effect on insulin resistance, cholesterol/lipid metabolism, or blood pressure [31,82,84,85,89,91]. However, in some cases, the weight loss was as low as 1–2% of initial body weight. Some of the observed differences might be due to the different genetic makeup of the researched population, differences in the IF/TRF protocols used, and differences in the time of day in which the eating period was set (circadian rhythms).

In conclusion, to establish the significance of fasting in controlling metabolic risk factors and the potential treatment of MetS, there is a need for more randomized control trials on larger cohorts of patients with MetS to gather higher yield evidence. Additionally, economic analysis is required to confirm the presumed cost-utility of IF/TRF.

## Figures and Tables

**Table 1:** Studies examining fasting on animal models of MetS and obesity.

Study	Cohort	Fasting Regime	Duration	Results
Hatori et al. [73]	Mice fed with a high-fat diet (HFD, 61% energy from fat)	16 h fast/ 8 h eating window	18 weeks	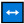 caloric intake ↓ body weight ↑ insulin sensitivity ↓ hepatosteatosis ↓ inflammation ↑ motor coordination ↑ CREB, mTOR, AMPK function
Sherman et al. [74]	Mice fed HFD ad libitum/mice fed HFD time-restricted	Fed during zeitgeber time 4 and 8 (zeitgeber time 0 is the time of lights on)	18 weeks	↓ body weight ↓ cholesterol levels ↓ TNF-α ↑ insulin sensitivity
Pak et al. [75]	Ad libitum mice/30%CR mice/30%CR in 12 h feed period/30% CR mice fed once in morning/ Ad libitum mice in 3-h feed period	12 h TRF/ Once in the morning feeding/ 3 h TRF	16 weeks	Fasting is needed for CR-induced improvements in glucose metabolism, frailty, and lifespan in C57BL/6J male mice
Li et al. [79]	Mice fed ad libitum chow diet/ mice on EODF	Every-other-day fasting (EODF)	30 days	↑ white adipose tissue browning ↓ obesity ↓ hepatic steatosis ↑ insulin sensitivityeffects exerted through shaping of the gut microbiota
Villanueva et al. [80]	Drosophila melanogaster (fruit fly) model of obesity	12 h TRF	3 weeks	↓ muscular fat deposits ↑ Phospho-AKT level ↑ insulin sensitivity ↓ mitochondrial aberrations
Munhoz et al. [81]	Young female Wistar rats	24 h fast/ 24 h of free access to the same chow	12 weeks	↓ weight gain ↓ food intake ↑ fat reserves ↑ plasma insulin concentrations ↓ muscle mass

Abbreviations: HFD = high-fat diet, CR = caloric restriction, CREB = cAMP response element-binding protein, mTOR = mammalian target of rapamycine, AMPK = 5’ AMP-activated protein kinase, TNFα = tumor necrosis factor alpha, TRF = time-restricted feeding, EODF = every-other-day fasting.

**Table 2:** Studies examining fasting in patients with cardiometabolic risk factors.

Study	Cohort	Fasting Regime	Duration	Results
Wilkinson et al. [31]	19 participants with MetS (13 male; 6 female)	14 h fast (from 8/10 a.m. to 6/8 p.m.)	12 weeks	↓ caloric intake ↓ body weight ↓ body fat and visceral fat ↓ waist circumference ↓ blood pressure ↓ cholesterol (total, LDL)
Parvaresh et al. [91]	69 participants with MetS (35 male; 34 female)	Modified ADF (Sat/Mon/Wed)	8 weeks	↓ body weight ↓ waist circumference ↓ systolic blood pressure ↓ fasting plasma glucose 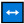 cholesterol (total, HDL, LDL) 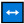 HOMA- IR
Sutton et al. [82]	8 participants (men with prediabetes); proof-of-concept study	eTRF eating period form 8 a.m. to 2 p.m. (while controlling for weight loss)	5 weeks	↓ insulin levels ↓ blood pressure ↓ oxidative stress ↑ insulin sensitivity
Gabel et al. [85]	46 participants with obesity (41 female, 5 male)	16/8 h fasting regime	12 weeks	↓ body weight ↓ energy intake ↓ systolic blood pressure 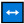 fat mass 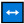 cholesterol 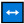 fasting glucose 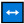 HOMA IR
Kesztyüs et al. [86]	40 participants with abdominal obesity (31 female, 9 male)	8–9 h TRF	12 weeks	↓ body weight ↓ waist circumference and waist–hip ratio
Chow et al. [89]	20 participants (17 female and 3 male)	16/8 h fasting regime	12 weeks	↓ reduced weight ↓ lean mass ↓ visceral fat 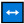 blood pressure 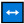 cholesterol 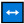 2 h oral glucose tolerance test
Lowe et al. [84]	116 overweight or obese adults (46 female, 70 male)	TRF (eating from 12–8 pm)	12 weeks	↓ body weight (in TRE group- compared to the starting weight) 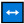 body weight between groups
Cienfuegos et al. [87]	58 obese adults (53 female, 5 male)	4 h TRF, 6 h TRF	8 weeks	Both regimens achieved: ↓ body weight, ↓ fasting insulin ↓ insulin resistance ↓ oxidative stress
Parr et al. [88]	19 participants with T2DM (10 female, 9 male)	9 h TRF	6 weeks	TRF compliance was achieved ~5 days/week 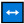 dietary intake 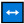 body weight 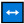 glycemic control

Abbreviations: ADF = alternate-day fasting, eTRF = early time-restricted feeding, TRE = time-restricted eating, HOMA-IR = Homeostatic Model Assessment for Insulin Resistance.

## Data Availability

Not applicable.
